# Herpesvirus and neurological manifestations in patients with severe coronavirus disease

**DOI:** 10.1186/s12985-022-01828-9

**Published:** 2022-06-08

**Authors:** Vanessa Cristine de Souza Carneiro, Soniza Vieira Alves-Leon, Dmitry José de Santana Sarmento, Wagner Luis da Costa Nunes Pimentel Coelho, Otacilio da Cruz Moreira, Andreza Lemos Salvio, Carlos Henrique Ferreira Ramos, Carlos Henrique Ferreira Ramos Filho, Carla Augusta Barreto Marques, João Paulo da Costa Gonçalves, Luciane Almeida Amado Leon, Vanessa Salete de Paula

**Affiliations:** 1 Laboratory of Molecular Virology, Oswaldo Cruz Institute/Fiocruz, Rio de Janeiro, Brazil; 2 Laboratory of Technological Development in Virology, Oswaldo Cruz Institute/Fiocruz, Rio de Janeiro, Brazil; 3 Department of Neurology/Reference and Research Center for Multiple Sclerosisand Other Central Nervous System Idiopathic Demyelinating Inflammatory Diseases, Clementino Fraga Filho University Hospital, Federal University of Rio de Janeiro, Rio de Janeiro, Brazil; 4 Department of Oral Diagnosis, School of Dentistry, State University of Paraíba, Araruna, PB Brazil; 5 Real Time PCR Platform RPT09A, Laboratory of Molecular Biology and Endemic Diseases, Oswaldo Cruz Institute/Fiocruz, Rio de Janeiro, Brazil; 6 Laboratory of Translacional Neurosciences, Biomedical Institute, Federal University of the State of Rio de Janeiro/UNIRIO, Rio de Janeiro, Brazil; 7 Laboratory of Molecular Virology, Oswaldo Cruz Institute, Oswaldo Cruz Foundation, 4365 Brasil Ave., Manguinhos, Rio de Janeiro, RJ 21040-360 Brazil

**Keywords:** SARS-CoV-2 infection, Herpesvirus, Neurological manifestations

## Abstract

**Background:**

Certain clinical manifestations of coronavirus disease (COVID-19) mimic those associated with human herpesvirus (HHV) infection. In this study, we estimated the prevalence of herpesvirus in patients with COVID-19 and determined if coinfection is associated with poorer outcomes and neurological symptoms.

**Methods:**

We analyzed samples of 53 patients diagnosed with COVID-19. The samples were evaluated for the presence of alphaherpesviruses, betaherpesviruses, and gammaherpesviruses, and the viral loads were quantified using quantitative polymerase chain reaction (qPCR) method.

**Results:**

Among the patients, in 79.2% had detection at least one type of herpesvirus. HHV-6 (47.2%), cytomegalovirus (43.3%), and HHV-7 (39.6%) showed the highest detection rates. Patients with a high severe acute respiratory syndrome coronavirus 2 (SARS-CoV-2) load were more likely to show herpes simplex virus 1 detection (*p* = 0.037). Among patients coinfected with SARS-CoV-2 and HHVs, 26.4% showed central nervous system-associated neurological symptoms and herpetic manifestations. A statistically significant association was observed between neurological changes and HHV-6 detection (*p* = 0.034).

**Conclusions:**

The findings showed a high prevalence of herpesvirus in patients with COVID-19. Furthermore, even though SARS-CoV-2 and HHV coinfection was not associated with poorer outcomes, the findings demonstrated the association between neurological symptoms and HHV-6 detection.

**Supplementary Information:**

The online version contains supplementary material available at 10.1186/s12985-022-01828-9.

## Background

People infected with severe acute respiratory syndrome coronavirus 2 (SARS-CoV-2) commonly present mild symptoms, such asfever, cough, and fatigue. However, in certaincases, the manifestations include pneumonia, multiple organ failure, and death [1]. Some patients with coronavirus disease (COVID-19) develop other clinical manifestations, such as cutaneous lesions and neurological disorders, in addition to respiratory symptoms [2, 3]. A significant percentage (80%) of patients develop neurological complications during SARS-CoV-2 infection, such asstroke, headache, dizziness, mental confusion, ageusia, anosmia, myelitis, and encephalitis [2, 4–6]. During the COVID-19 pandemic, cases of COVID-19 with human herpesvirus (HHV) reactivation were reported, which were related to lymphocytopenia owing to SARS-CoV-2 infection, or even to drug reactions [7, 8]. Studies have suggested that patients with neurological manifestations of SARS-CoV-2 infection should undergo detection tests for opportunistic neurotropic viruses, such as HHVs, since therapeutic strategies are available for such infections, which can help reduce morbidity and mortality or improve disease prognosis [9, 10]^.^

To date, the issue of neurological symptoms in patients with COVID-19 has been discussed extensively [5]. Studies have shown that SARS-CoV-2 can infect cells of the nervous system, even neurons with low expression of angiotensin converting enzyme-2, which plays an important role in viral entry [6, 10, 11]. Nervous system involvement appears to occur early, and the neurofilament content is elevated in patients with more severe outcomes [12]. However, other studies in patients with COVID-19 and neurological disorders have reported low levels or absence of viral RNA in the cerebrospinal fluid (CSF) [13, 14]. Therefore, the association between SARS-CoV-2 and neurological disorders is still not well understood [6, 15].

Although neurological disorders have been associated with infections caused by other opportunistic and neurotropic viruses [16], HHV coinfections in patients with COVID-19 remain poorly investigated. There are nine HHV species: human alphaherpesvirus 1 (herpes simplex virus (HSV)-1), human alphaherpesvirus 2 (HSV-2), human alphaherpesvirus 3 (varicella-zoster virus (VZV)-3), human gammaherpesvirus 4 (Epstein–Barr virus, EBV), human betaherpesvirus 5 (cytomegalovirus, CMV), human betaherpesvirus 6A, 6B, and 7 (HHV-6A, HHV-6B, and HHV-7), and human gammaherpesvirus8 (HHV-8) [17]. All of the viruses are double-stranded DNA viruses belonging to family *Herpesviridae* and are capable of establishing latency in their hosts, with the potential for reactivation in immunocompromised patients, which can lead to several complications [18, 19]. Neurological disorders have been reported in patients with herpesvirus reactivation [20]. HHV-1 is considered the main cause of viral encephalitis, which is associated with epileptic seizures in most cases [21]. Futhermore, HHV-1 has been reported as a trigger for NMDA receptor autoimmune encephalitis, as recently reported in a patient after SARS-CoV-2 infection [22] which could be attributed to molecular mimicry between the SARS-CoV-2 and NMDA receptors [23]. Except for HHV-8, all herpesviruses can cause encephalitis [21, 24, 25]. Furthermore, active infection by HHVs may also be associated with myelitis, stroke, and transient ischemic attack [26–28].

Owing to the high HHV prevalence worldwide and the complex course of infection, characterized by lifelong infection with periods of latency interspersed with periodic episodes of reactivation, the diagnosis of coinfections is necessary to help establish potential associations with neurological manifestations, which may affect the prognosis of SARS-CoV-2-infected patients.

This is the first study to investigate the frequency of the detection of all types of herpesviruses in critically ill patients with COVID-19, and thus, to evaluate if SARS-CoV-2 infection could be a risk factor for *Herpesviridae* reactivation. In addition, we aimed to investigate the association between HHV detection and neurological manifestations in patients with COVID-19.

## Materials and methods

### Patients and samples

In this study, we analyzed nasopharyngeal swab samples collected from 53 patients admitted to the intensive care unit of the Clementino Fraga Filho University Hospital (HUCFF) located in Rio de Janeiro City, Brazil. All patients underwent follow-up till they were discharged. The study was approved by the Ethics Committee of the HUCFF (Protocol Number 4.103.725). The inclusion criteria were: (1) patients with COVID-19 diagnosis confirmed by RT-PCR [29] and (2) availability of consent forms signed by the patient or guardian/legal representative. Pregnant women were excluded from the study. The clinical evolution and demographic data of patients were collected from medical records. The assessment and interpretation of the findings of neurological examinations were reviewed by neurologists who collaborated in the study.

### Virus detection

Viral DNA was extractedfrom the nasal swab samples usinga viral DNA extraction kit (Qiagen, Valencia, CA, USA), according to the manufacturer's recommendations. The viral loads of alphaherpesviruses (HSV-1, HSV-2, and VZV), betaherpesviruses (CMV, HHV-6, and HHV-7), and gammaherpesviruses (EBV and HHV-8) were quantified using real-time TaqMan PCR, as described previously [30–34] (the sequences primers, probe and standard curve are in Additional file 1).

### Statistical analysis

Kendall's Tau-b Correlation was used to determine the correlation between the COVID-19 load viral with the herpesvirus load viral to determine the association of the variables corticosteroid use, lymphocytopenia, outcomes, and neurological symptoms with herpesvirus detection, Pearson's Chi-square or Fisher's exact tests were used, and the viral load was evaluated using the Mann–Whitney test (Kolmogorov–Smirnov, *p* < 0.05). A 5% significance level was used. Statistic alanalyses were performed using SPSS version 20.0 (SPSS, Inc., Chicago, IL, USA).

## Results

All 53 patients diagnosed and hospitalized with COVID-19 underwent follow-up from hospital evolution till the outcome (discharge or death). The average hospital stay was of 24.36 ± 16.65 days, ranging from 3 to 70 days. Most patients were male (50.9%), with age ranging between 17 and 95 years, and a mean age of 63.51 ± 15.68 years. Patients were classified using the Sequential Organ Failure Assessment (SOFA) score (a SOFA score > 9 was considered to represent severe cases). Among the patients, 39.6% were considered to have severe disease (SOFA > 9), and 58.5% of the patients evolved to hospital discharge. Almost all patients (98.1%) had at least one comorbidity, the most common ones being systemic arterial hypertension, diabetes mellitus, and chronic kidney disease (Table 1). Regarding on COVID-19 symptoms treatment, the study population was predominantly treated with corticosteroids (33/53), while only 1/53 were treated with Interferon B1, and 2/53 with antibiotics.

**Table 1:** Clinical and demographic characteristics of patients admitted to the intensive care unit

Variable	N	%
*Gender*		
Male	27	50.9
Female	26	49.1
*Pulmonary impairment*		
< 10%	20	37.8
10–50%	27	50.9
> 50%	6	11.3
*Support O*_*2*_		
None	12	22.6
Nasal catheter	16	30.2
Orotracheal intubation	25	47.2
*Outcome*		
Discharge	31	58.5
Death	22	41.5
*SOFA score*		
≤ 9 (mild to moderate)	32	60.4
> 9 (severe)	21	39.6
*Comorbidities*^a^		
Yes	52	98.1
No	1	1.9
Total	53	100
SOFA = Sequential Organ Failure Assessment Comorbidities^a^		
Systemic arterial hypertension—81.1% (43/53)		
Diabetes mellitus—45.3%(24/53)		
Chronic kidney disease—24.5% (13/53)		
Cardiovascular disease—20.8% (11/53)		
Pulmonary disease—11.3% (6/53)		
Immunodeficiency—11.3% (6/53)		
Maglinities—7.5% (4/53)		

^a^The patient may have had more than one comorbidity

With respect to the prevalence of herpesviruses, 79.2% (42/53) of the patients tested positive for at least one herpesvirus. Of the 42 patients, 75.0% showed coinfection with two or more viral subtypes. The most prevalent herpes viruses were HHV-6 (47.2%), CMV (43.4%), HHV-7 (39.6%), and HHV-8 (17%). The HSV-1 load correlated with the SARS CoV-2 load (*p* = 0.037). The other correlations were weak and without statistical association, with positive tests for CMV, HHV-7 and HHV-8 and negative tests for EBV and HHV-6 (Table 2). The outcomes and viral load were not found to be associated with herpesvirus detection (using the Mann–Whitney test, *p* > 0.05). Additionally, we evaluated the association of corticosteroid use and lymphocytopenia with herpesvirus detection (Pearson's Chi-squareor Fisher's exacttest, *p* > 0.05), but no statistically significant associations were found between any variable and herpesvirus detection (Table 3).

**Table 2:** Prevalence of herpesvirus reactivation, viral load, and correlation of herpesvirus reactivation with the SARS-CoV-2 load

Virus	Prevalence n (%)	SARS-CoV-2 load (mean ± DP)	Kendall’s Tau-b Correlation coefficient (coefficient; *p* value)
HSV-1	9/53 (17)	3.54E+9 ± 1.0E+10	0.556; *p* = 0.037*
HSV-2	1/53 (1.9)	1930	–
EBV	15/53 (28.3)	1.52E+12 ± 3.99E+12	− 0.162; *p* = 0.400
CMV	23/53 (43.4)	1.31E+11 ± 3.28E+11	0.020; *p* = 0.895
VZV	4/53 (7.5)	1.32E+10 ± 2.26E+10	–
HHV-6	25/53 (47.2)	8.52E+11 ± 2.14E+12	− 0.223; *p* = 0.102
HHV-7	21/53 (39.6)	2.83E+11 ± 6.57E+11	0.181; *p* = 0.251
HHV-8	9/53 (17)	9.50E+5 ± 2.40E+6	0,355; *p* = 0.139

^a^Kendall’s Tau-b coefficient indicates the association of the SARS-CoV-2 load with the load viral of each herpesvirus. Missing values indicate that the test could not be conducted owing to a small sample size

*SARS-CoV-2* severe acute respiratory syndrome coronavirus 2, *HSV* herpes simplex virus, *VZV* varicella zoster virus, *EBV* Epstein–Barr virus, *CMV* cytomegalovirus, *HHV* human herpesvirus

*Statistical significance

**Table 3:** Association of corticosteroid use and lymphocytopenia with herpesvirus reactivation

Viral type	Corticosteroid use		*p*	Lymphocytopenia		*p*	Total
	Yes N (%)	No N (%)		Yes N (%)	No N (%)		
*HSV-1*							
Yes	8 (88.9)	1 (11.1)	0.129^a^	6 (66.7)	3 (33.3)	0.474^a^	9 (100)
No	25 (56.8)	19 (43.2)		22 (50)	22 (50)		44 (100)
*HSV-2*							
Yes	1 (100)	0 (0)	1.000^a^	1 (100)	0 (0)	1.000^a^	1 (100)
No	32 (61.5)	20 (38.5)		27 (51.9)	25 (48.1)		52 (100)
*EBV*							
Yes	11 (73.3)	4 (26.7)	0.296^b^	10 (66.7)	5 (33.3)	0.205^b^	15 (100)
No	22 (57.9)	16 (42.1)		18 (47.4)	20 (52.6)		38 (100)
*CMV*							
Yes	12 (52.2)	11 (47.8)	0.185^b^	11 (47.8)	12 (52.2)	0.523^b^	23 (100)
No	21 (70)	9 (30)		17 (56.7)	13 (43.3)		30 (100)
*VZV*							
Yes	2 (50)	2 (50)	0.627^a^	3 (75)	1 (25)	0.613^a^	4 (100)
No	31 (63.3)	18 (36.7)		25 (51)	24 (49)		49 (100)
*HHV-6*							
Yes	14 (56)	11 (44)	0.374^b^	12 (48)	13 (52)	0.506^b^	25 (100)
No	19 (67.9)	9 (32.1)		16 (57.1)	12 (42.9)		28 (100)
*HHV-7*							
Yes	14 (66.7)	7 (33.3)	0.592^b^	8 (381)	13 (61.9)	0.082^b^	21 (100)
No	19 (59.4)	13 (40.6)		20 (62.5)	12 (37.5)		32 (100)
*HHV-8*							
Yes	5 (55.6)	4 (44.4)	0.715^b^	2 (22.2)	7 (77.8)	0.087^b^	9 (100)
No	28 (63.6)	16 (36.4)		26 (59.1)	18 (40.9)		44 (100)

*HSV* herpes simplex virus, *VZV* varicella zoster virus, *EBV* Epstein–Barr virus, *CMV* cytomegalovirus, *HHV* human herpesvirus

^a^Fisher's exact test; ^b^Pearson's Chi-square test

The occurrence of neurological symptoms was investigated in patients with COVID-19. Symptoms related to the CNS were detected in 26.4% of the patients, where symptoms related to the PNS where observed in 7.5% of the patients (Table 4). CNS symptoms were more prevalent in patients with herpesvirus detection, with statistically significant values obtained for HHV-6 (40% in patients with HHV-6 detection vs 14.3% in patients without HHV-6 detection). Of note, there was detection the CMV and HHV-7 in multiple patients with neurological symptoms, but the findings were not of statistical significance. HSV-1, HSV-2, VZV, HHV-8 and EBV were also detected in patients who showed CNS-associated neurological symptoms. There was no association of herpesvirus detection with PNS-related symptoms (Table 5). We looked for an association betewn neurological changes together with demographic data such as age and sex, but we did not find a significant association. The age variable, it presented a value of *p* = 0.809 for the CNS and *p* = 0.961 for the SNP. The sex variable presented a value of *p* = 0.480 for the CNS and *p* = 0.999 for the SNP. The statistical analysis of the data also showed that there were no significant association between neurological disorders with comorbidities and the use of corticosteroids.

**Table 4:** Frequency of neurological symptoms

Symptoms	n	%
*Central nervous system*^a^	14/53	26.4
Impaired consciousness	8/53	15.1
Headache	5/53	9.4
Dizziness	1/53	1.9
Acute cerebrovascular disease	1/53	1.9
Seizure	1/53	1.9
*Peripheral nervous system*^a^	4/53	7.5
Hypo/ageusia	2/53	3.8
Hypo/anosmia	1/53	1.9
Vision impairment	1/53	1.9
Peripheral neuropathy	1/53	1.9

^a^The patient may have had more than one symptom

**Table 5:** Association of neurological symptoms with herpesvirus reactivation

Virus	Central nervous system		*p*	Peripheral nervous system		*p*	Total
	Yes N (%)	No N (%)		Yes N (%)	No N (%)		
*HSV-1*							
Yes	3 (33.3)	6 (66.8)	0.684^a^	2 (22.2)	7 (77.8)	0.129^a^	9 (100)
No	11 (25)	33 (75)		2 (4.5)	42 (95.5)		44 (100)
*HSV-2*							
Yes	1 (100)	0 (0)	0.264^a^	0 (0)	1 (100)	0.999^a^	1 (100)
No	13 (25)	39 (75)		4 (7.7)	48 (92.3)		52 (100)
*EBV*							
Yes	5 (33.3)	10 (66.7)	0.504^a^	0 (0)	15 (100)	0.568^a^	15 (100)
No	9 (23.7)	29 (76.3)		4 (10.5)	34 (89.5)		38 (100)
*CMV*							
Yes	9 (39.1)	14 (60.9)	0.066^b^	0 (0)	23 (100)	0.124^a^	23 (100)
No	5 (16.7)	25 (83.3)		4 (13.3)	26 (86.7)		30 (100)
*VZV*							
Yes	2 (50)	2 (50)	0.282^a^	0 (0)	4 (100)	0.999^a^	4 (100)
No	12 (24.5)	37 (75.5)		4 (8.2)	45 (91.8)		49 (100)
*HHV-6*							
Yes	10 (40)	15 (60)	0.034^b*^	0 (0)	25 (100)	0.113 ^a^	25 (100)
No	4 (14.3)	24 (85.7)		4 (14.3)	24 (85.7)		28 (100)
*HHV-7*							
Yes	8 (38.1)	13 (61.9)	0.118^b^	1 (4.8)	20 (95.2)	0.999^a^	21 (100)
No	6 (18.8)	26 (81.2)		3 (9.4)	29 (90.6)		32 (100)
*HHV-8*							
Yes	3 (33.3)	6 (66.7)	0.684^b^	0 (0)	9 (100)	0.999^a^	9 (100)
No	11 (25.0)	33 (75.0)		4 (9.1)	40 (90.9)		44 (100)

*SARS-CoV-2* severe acute respiratory syndrome coronavirus 2, *HSV* herpes simplex virus, *VZV* varicella zoster virus, *EBV* Epstein–Barr virus, *CMV* cytomegalovirus, *HHV* human herpesvirus

*Statistical significance

^a^Fisher's exact test; ^b^Pearson's Chi-square test

## Discussion

To our knowledge, this study is the first to provide a broad overview of all HHV infections in patients with severe COVID-19. The detection the at least one type was observed in 42 patients, whereas some patients showed the detection of up to four types of herpesviruses concurrently. These data indicate that the state of immunosuppression in SARS-CoV-2 infection, characterized by symptoms such as lymphocytopenia, may possibly trigger a cycle of opportunistic virus reactivations, which makes it necessary to monitor the influence of these viruses on the course of COVID-19 [10, 35]. These findings reiterate the importance of further studies on family *Herpesviridae* that investigate their reactivation frequency in the population and interference with other pathogens [36]. Interestingly, we detected HHV-8 in 17% of patients. Previous studies have observed the low prevalence of HHV-8 in the Brazilian population compared to other herpesviruses [37]. However, a considerable detection rate of HHV-8 in our study cohort suggests that the prevalence of HV-8 may be underestimated by serological detection methods curreent.

Some studies have shown the high incidence of herpetic reactivation in patients with COVID-19 severe, showing that reactivation herpesvirus is associated with aprolonged ICU stay and respiratory support [35, 38]. However, despite the high level of herpesvirus detection in our study, only HSV-1 showed a viral load correlation with SARS CoV-2 (*p* = 0.037) (Table 2), indicating that patients with a high viral load of SARS-CoV-2 tend to show HSV-1 reactivation. These data represent an important finding. According to some reported cases, patients coinfected with SARS-CoV-2 and HSV-1 can develop complications such as acute liver failure, neurological symptoms, and septic shock [9, 10, 39]. This information highlights the importance of the early screening of HSV-1 reactivation in patients with COVID-19-related complications and the necessity of appropriate treatment and avoidance of worse outcomes. However we did not find any significant association between the detection of other herpesviruses and worse outcomes, as reported in previous studies [35, 38, 40].

In hospitalized patients with COVID-19, a high prevalence of some viral subtypes of *Herpesviridae*, such as HSV-1 and EBV, has been reported [35, 41]. In our study, both HSV-1 and EBV were detected in 17% and 28.3% of the patients, respectively; however, the betaherpesviruses HHV-6, CMV, and HHV-7 showed the highest DNA loads.

Although our study population showed a high rate of herpesvirus detection, a limitation of the study was the lack of information about the exact date of symptom onset and the time of sample collection. Seeßle et al. [41] recently showed that the HSV-1 reactivation rate increased 11 days after the onset of symptoms in patients with COVID-19; therefore, possibly, there activation rate of some herpesviruses may be higher than that observed in our study, because the time between symptom onset and sample collection may vary.

In 26.4% of the patients, we observed CNS-associated neurological symptoms, such as impaired consciousness, headache, dizziness, acute cerebrovascular disease, and seizure. Similarly, a recent study confirmed SARS-CoV-2/HSV-1 coinfection in a patient with loss of consciousness, disorientation, and dizziness [9], which are symptoms suggestive of herpetic infection. EBV reactivation has also been associated with persistent symptoms observed in patients with COVID or long COVID,including some neurological manifestations, such as confusion and headache, which were observed in our patient group as well [42]. The most commonly detected herpesviruses in patients with neurological changes were HHV-6, CMV, and HHV-7. Although a statistically significant association between neurological changes and herpesvirus detection was only observed for HHV-6, CMV was also detected in several patients, and the reactivation of this virus is also associated with neurological changes in immunocompromised patients [20]. The immunosuppressive condition triggered by SARS-CoV-2 infection may play a role in reactivation herpesvirus, which may cause diffuse encephalitisand myelitis [20]. In addition, some patients were treated with corticosteroids, and we know that use of corticosteroids can trigger herpesvirus reactivation, studies have demonstrated the association between the use of corticosteroids and reactivation of CMV and HHV-6, which coincidentally also were the most detected herpesviruses in our study population [43]. Furthermore, our findings also suggested an association between neurological changes and HSV-1 detection in patients with a high SARS-CoV-2 load. In this group of patients, 33.3% (3/9) showed changes in the CNS and 22.2% (2/9) in the PNS. Given the strong association between HSV-1 reactivation and neurological changes, further investigation of this association is necessary.

Previous studies also corroborate with our findings, such as, Jumah et al. [44] that reported a case of neurological disorder caused by reactivation of HHV-6 during COVID-19, in which the patient improved after adequate treatment. The findings of this case provided evidence of HHV-6 reactivation in the CNS associated with the immunocompromised status acquired during SARS-CoV-2 infection. HHV-6 has a high prevalence in the population and are neurotropic, causing neurological disorders such as dizziness, epilepsy, and encephalitis [27, 45]. Increasing evidence has indicated the association of HHV-6 infection with various neurological alterations in immunocompromised and immunocompetent individuals [46, 47]. The findings of this case provided evidence of HHV-6 reactivation in the CNS associated with the immunocompromised status acquired during SARS-CoV-2 infection [44]. HHV-6 has a high prevalence in the population and are neurotropic causing neurological disorders such as dizziness, epilepsy, and encephalitis [27, 45]. Increasing evidence has indicated the association of HHV-6 infection with various neurological alterations in immunocompromised and immunocompetent individuals [46, 47]. Based on our findings and the ability of HSV-1 and HHV-6 to cause neurological disorders during reactivation, we suggest that this viral subtype should be investigated as a possible cause of neurological changes in SARS-CoV-2-infected individuals.

However, although the detection of herpesvirus in body fluids of patients in an immunosuppressed state is strongly suggestive of a reactivation, we cannot determine if that it is a reactivation or a primary infection. Although, it has been shown that the global prevalence of some herpesviruses is high, between 80- 90% [48], which includes the herpesviruses detected with high prevalence in our study cohort: Adane and Getawa [49], reported a global seroprevalence of CMV in blood donors of 83.16%, and Souza et al. [50], in Brazil showed a seroprevalence of 96.4%. As for HHV-6 and 7, both are highly prevalent in the global population, and specific IgG antibodies for HHV-6 and to HHV-7 were detected in more than 90% of adults [51]. For HHV-6, Linhares et al. [52], detected a seroprevalence in north-eastern Brazilian population of 76.5%. Subsequently, Freitas and Linhares [53], detected a seroprevalence of HHV-6 in northern Brazilian population of 90%. These data show that although be scarce studies investigating the prevalence of the herpesviruses in the Brazilian population, we can consider them to be highly prevalent viruses, which strengthens the hypothesis that the high prevalence found in our study be caused by viral reactivation.

A limitation of the study that should be considered, is the size of the study cohort, and the inability of examining the CSF for viral DNA because of the clinical status of the patients, and the overload on the public health system during the peak of the pandemic. However, we highlight that our cohort are from patients hospitalized in the intensive care unit with severe COVID-19, and our sampling was well characterized with full information about clinical data and the course of the infection. We consider these information highly relevant and unusual, since most published study about herpesvirus reactivation in patients with covid-19 are case reports or with a study population smaller than ours [9, 10, 35, 38]. To date, there are no reports of other studies that investigate the detection of all herpesviruses in patients with COVID-19 considering cases mild, moderate and severe. We believe that despite small sample size, the present study is relevant to stimulate the investigation of other opportunistic viruses, such as herpesviruses in the context of COVID-19.

Regarding on, COVID-19 infection as a risk factor for HHV reactivation, in our study we did not aim to demonstrate that COVID-19 infection could be a risk factor for Herpesvirus reactivation, instead, based on our findings together with previous report [44], we hypothesized that the immunosuppressive condition triggered by SARS-CoV-2 infection could lead to HHVs reactivation, as well as the infection treatment with corticosteroids, but experimental studies are needed to clear this.Corroborating this, Chen et al. [54] showed that patients with severe covid-19 present a state of immunosuppression, facilitating the occurrence co-infection caused by opportunistic infectious agents. Le Balc'h et al. [38] in their study population of patients with severe COVID-19, found reactivation of CMV and HSV1, although they did not investigate other herpesviruses.

A limitation of this study was that the size of the study population may have interfered with the result of the association between lymphocytopenia and the use of corticosteroids, since corticosteroids, such as steroids and immunomodulatory drugs, have been identified as triggers for the reactivation of latent herpesviruses in the host [7, 55]. Our study population predominant was treated with corticosteroids 33/53, only 1/53 were treated with Interferon B1 and 2/53 with antibiotcs, however we have no information on the duration the treatment.

We believe that a major contribution of our study is to alert clinician to the risk of neurological manifestations during COVID-19, that could be associated with other pathogens that have specific treatment, such as HHVs, and to encourage the differential diagnosis, due to the high rate of herpesvirus detection in patients with COVID-19.

## Conclusion

In this study, we reported a high prevalence (79.2%) of herpesvirus detection in patients with COVID-19. We also showed the association between HHV-6 detection and neurological disorders. Our findings showed that HHV detection may be underestimated, and that herpesviruses other than HSV-1, EBV, and CMV may also be associated with neurological manifestations. The results highlight the importance of investigating the role of opportunistic viruses, such as herpesviruses, in the context of COVID-19, and their influence on the prognosis and neurological manifestations in patients infected with SARS-CoV-2. In addition, future investigations should focus on the role of herpesviruses in modulating the immune system via the regulation of gene expression during SARS-CoV-2 infection in critically ill patients, since herpesviruses harbor several mechanisms for regulating the host immune system.

## Supplementary Information

**Additional file 1.** Sequence of primers, probes and synthetic standard curve of herpesviruses.

## Data Availability

The data analyzed during the study are available from the corresponding author on reasonable request.

## Abbreviations

COVID-19: Coronavirus disease
HHV: Human herpesvirus
qPCR: Quantitative polymerase chain reaction
SARS-CoV-2: Severe acute respiratory syndrome Coronavirus-2
CSF: Cerebrospinal fluid
RNA: Ribonucleic acid
HSV: Herpes simplex vírus
CMV: Cytomegalovirus
EBV: Epstein–Barr vírus
VZV: Varicella-zoster virus
HUCFF: Clementino Fraga Filho University Hospital
RT-PCR: Real-time reverse transcription-polymerase chain reaction
DNA: Deoxyribonucleic acid
SOFA: Sequential Organ Failure Assessment
CNS: Central nervous system
PNS: Peripheral nervous system
ICU: Intensive care unit
