# Supplementary material for: Herpesvirus and neurological manifestations in patients with severe coronavirus disease
Source: Virol J. 2022 Jun 8;19:101. doi: 10.1186/s12985-022-01828-9 (PMC9174631; doi:10.1186/s12985-022-01828-9)
Supplement: Supplementary file 1 — Additional file 1. Sequence of primers, probes and synthetic standard curve of herpesviruses. [file 12985_2022_1828_MOESM1_ESM.pdf]

Additional file 1. Sequence of primers, probes and synthetic standard curve of herpesviruses

virus	Probe (5'-3')	Primers sense (5'- 3')	Primers anti-sense (5 -3')	Standard curve	Reference
HSV-1	CCCGCGCACCACGTC	CCAACTGCACCATGATCATCGA	GATGTTTGTCACCGCAACGAA	TTCGTGATGTTTGTCACCGCAACGAACGTATGACGTGGTGCGCGGATGCGTTCGATGATCATGGTGCAGTTGGCTGATA	Lima et al., 2017
HSV-2	CGCGGAGACATTCGAGTACCAGATCG	CGCTCTCGTAAATGCTTCCCT	TCTACCCACAACAGACCCACG	ATGCTATCTACCCACAACAGACCCACGTACGTACGATCTGGTACTCGAATGTCTCCGCGCATGCAGGGAAGCATTTACGAGAGCGCTGATC	Lima et al., 2017
VZV	ATTCAGCAATGGAAACACACGACGCC	CGGCATGGCCCGTCTAT	TCGCGTGCTGCGGC	CGATATCGCGTGCTGCGGCGCTATGGCGTCGTGTGTTTCCATTGCTGAATCGTGCATAGACGGGCCATGCCGTACTG	Carneiro et al., 2022
EBV	TGCAGCTTTGACGATGG	CCGCTCCTACCTGCAATATCA	GGAAACCAGGGAGGCAAATC	TTCGTGGAAACCAGGGAGGCAAATCCGTATCCATCGTCAAAGCTGCAATGCGTTGATATTGCAGGTAGGAGCGGCTGATA	Fellner et al., 2014
CMV	CCGTATTGGTGCGCGATCTGTTCAA	GGCCGTTACTGTCTGCAGGA	GGCCTCGTAGTGAAAATTAATGGT	TTCGTGGCCTCGTAGTGAAAATTAATGGTCGTATTTGAACAGATCGCGCACCAATACGGATGCGTTCCTGCAGACAGTAACGGCCCTGATA	Raposo et al., 2019
HHV-6	TTAGATGGTGGTGAGCTGGGATCGGT	AAAGACCTAAATTGCCGCTACCT	GCAAGCTCATGAACATCGTCA	TTCGTGCAAGCTCATGAACATCGTCACGTATACCGATCCCAGCTCACCACCATCTAAATGCGTAGGTAGCGGCAATTTAGGTCTTTCTGATA	Raposo et al., 2019 and Raposo et al., 2020
HHV-7	CTCGCAGATTGCTTGTTGGCCATG	CGGAAGTCACTGGAGTAATGACAA	CCAATCCTTCCGAAACCGAT	TTCGTCCAATCCTTCCGAAACCGATCGTATCATGGCCAACAAGCAATCTGCGAGATGCGTTTGTCATTACTCCAGTGACTTCCGCTGATA	Raposo et al., 2019 and Raposo et al., 2020
HHV-8	AGATCAAGTTCCGCCATAT	GGGCCCCGGATGATGTA	GCCCCATAAATGACACATTGG	TTCGTGCCCCATAAATGACACATTGGCGTATATATGGCGGAACTTGATCTATGCGT TACATCATCCGGGGCCCCTGATA	de Oliveira et al., 2020

Sequence of primers, probes and synthetic standard curve used in quantified using real-time PCR
